# Phylogenetic Classification of Global Porcine Deltacoronavirus (PDCoV) Reference Strains and Molecular Characterization of PDCoV in Taiwan

**DOI:** 10.3390/v13071337

**Published:** 2021-07-11

**Authors:** Fu-Chun Hsueh, Feng-Yang Hsu, Yu-Hsuan Chen, Hsing-Chun Shih, Wei-Hao Lin, Cheng-Yao Yang, Chuen-Fu Lin, Ming-Tang Chiou, Chao-Nan Lin

**Affiliations:** 1 Animal Disease Diagnostic Center, College of Veterinary Medicine, National Pingtung University of Science and Technology, Pingtung 91201, Taiwan; jim820723@gmail.com (F.-C.H.); fyhsu0328@gmail.com (F.-Y.H.); shing510183@gmail.com (H.-C.S.); whlin@g4e.npust.edu.tw (W.-H.L.); 2 Department of Veterinary Medicine, College of Veterinary Medicine, National Pingtung University of Science and Technology, Pingtung 91201, Taiwan; s88106s88106@gmail.com (Y.-H.C.); cflin2283@mail.npust.edu.tw (C.-F.L.); 3 Graduate Institute of Veterinary Pathobiology, College of Veterinary Medicine, National Chung-Hsing University, Taichung 40227, Taiwan; yangchengyao@nchu.edu.tw

**Keywords:** porcine deltacoronavirus (PDCoV), whole-genome sequencing, phylogenetic analysis, Taiwan

## Abstract

Porcine deltacoronavirus (PDCoV), a highly transmissible intestinal pathogen, causes mild to severe clinical symptoms, such as anorexia, vomiting and watery diarrhea, in piglets and/or sows. Since the first report of PDCoV infection in Hong Kong in 2012, the virus has readily disseminated to North America and several countries in Asia. However, to date, no unified phylogenetic classification system has been developed. To fill this gap, we classified historical PDCoV reference strains into two major genogroups (G-I and G-II) and three subgroups (G-II-a, G-II-b and G-II-c). In addition, no genetic research on the whole PDCoV genome or spike gene has been conducted on isolates from Taiwan so far. To delineate the genetic characteristics of Taiwanese PDCoV, we performed whole-genome sequencing to decode the viral sequence. The PDCoV/104-553/TW-2015 strain is closely related to the G-II-b group, which is mainly composed of PDCoV variants from China. Additionally, various mutations in the Taiwanese PDCoV (104-553/TW-2015) strain might be linked to the probability of recombination with other genogroups of PDCoVs or other porcine coronaviruses. These results represent a pioneering phylogenetic characterization of the whole genome of a PDCoV strain isolated in Taiwan in 2015 and will potentially facilitate the development of applicable preventive strategies against this problematic virus.

## 1. Introduction

Porcine deltacoronavirus (PDCoV) belongs to the genus *Deltacoronavirus*, family *Coronaviridae* and order *Nidovirales*. PDCoV consists of a positive-sense, single-stranded RNA virus, approximately 25.4 kb in length [1]. PDCoV was first detected in rectal swabs in Hong Kong in 2012 and has rapidly disseminated to the United States [2], Canada [3], China [4], Thailand [5], Vietnam [6], Laos [6], South Korea [7], Japan [8], Taiwan [9] and Mexico [10]. However, specific criteria for the genetic classification of PDCoV strains have been defined to date. Clinical signs of PDCoV include vomiting, anorexia, dehydration, and watery diarrhea in piglets [11,12]. Given that genetic insertions and deletions potentially altering the virulence or infectious pattern of porcine coronavirus have been discovered in Taiwan [13], analysis of the genetic composition of PDCoV might play a pivotal role in the development of preventive strategies to mitigate economic losses caused by outbreaks of this infectious disease.

The PDCoV genome encodes seven open reading frames (ORFs). ORF 1a/b, which is responsible for viral replication; spike (S), envelope (E) and membrane (M), which are responsible for viral assembly and induction of virus-specific neutralizing antibodies. Nonstructural protein 6 (NSP6) and nucleocapsid (N) are associated with binding abilities, and nonstructural protein 7 (NSP7) acts as an accessory function [14,15]. Specifically, the receptor-binding S protein is involved in viral entry and virus-host interactions [16,17]. Structural dissection of the PDCoV/USA/Ohio137/2014 strain reveals that the S protein comprises two primary regions: receptor-binding S1 subunits (aa 1-552) and membrane fusion S2 subunits (aa 553-1077) [18]. Recently, N-glycosylation sites and B-cell antigenic epitopes have been predicted in S and N proteins, respectively [19,20]. Previous investigations noted that PDCoV strains potentially coexisted with porcine epidemic diarrhea virus (PEDV), given that the first outbreaks of this virus also occurred during late 2014 to early 2015 [21,22]; however, genetic research on the whole genome or spike gene has not been conducted in Taiwan thus far.

Recently, PDCoV has become a capricious epidemic, which is frequently eclipsed by PEDVs [21], whilst mortality and economic losses caused by PDCoV pose an increasingly grave threat to the swine industry. To genetically characterize this virus, we performed whole-genome sequencing, and numerous specific aa mutations were identified in the PDCoV strain circulating in Taiwan in 2015. We also generated new criteria to classify previously identified global PDCoV strains based on whole-genome and spike protein phylogenies. The present study provides alarming results related to the persistence of PDCoV in the field and the potential for recombination with other prevalent porcine coronaviruses.

## 2. Materials and Methods

### 2.1. Sample Collection and Preliminary Examination by Real-Time PCR

A total of 35 intestinal and/or fecal specimens from piglets or sows with intermittent vomiting and watery diarrhea derived from 18 different pig farms were collected in 2015 and analyzed at the Animal Disease Diagnostic Center (ADDC) at the National Pingtung University of Science and Technology (NPUST). All these specimens were examined for PDCoV positivity by means of quantitative reverse transcription polymerase chain reaction (RT-qPCR), as previously reported [22]. However, quantification cycle (Cq) values of PDCoV in 91.42% (32/35) of our specimens were greater than 35.6, indicating that PDCoV loads were only 2 log_10_ (copies/μL) [22]. Three unique clinical samples were tested and exhibited fairly low Cq values of approximately 20 of PDCoV. All three sequences were collected from the same pig herd; thus, we chose one specimen for whole-genome sequencing.

### 2.2. Whole-Genome Sequencing

Viral total nucleic acid was extracted using the MagNA Pure LC total nucleic acid isolation kit (Roche Diagnostics, Mannheim, Germany), and the PrimeScript™ RT reagent kit (Takara Bio Inc., Kusatsu, Shiga, Japan) was used to synthesize complementary DNA (cDNA) following the manufacturer’s instructions. For whole-genome sequencing, 2 μL of template cDNA was first amplified by PCR using KAPA HiFi HotStart ReadyMix (Roche) and 17 PDCoV-specific primer pairs (Table S1) under the following thermal conditions: initial denaturation at 95 °C for 3 min; 35 cycles of 98 °C for 20 s, 58–64 °C for 15 s and 72 °C for 2 min and final extension at 72 °C for 1 min. Each PCR product was subjected to 1.5% agarose gel electrophoresis and visualized by ultraviolet illumination after ethidium bromide staining. The target nucleotide sequences were determined in both orientations using an autosequencer (ABI3730XL, Foster City, CA, USA).

### 2.3. Phylogenetic and Recombination Analysis

Multiple sequence alignment of the local PDCoV strain and historically global PDCoV reference strains were first constructed using Clustal W in the Molecular Evolutionary Genetics Analysis (MEGA) software, version 7 software program; then, phylogenetic trees were built through maximum likelihood estimation (MLE) in the Kimura 2-parameter (K2P) model, as previously described [13]. For the recombinant analysis, the SimPlot 3.5.1 software program was used to identify the potential recombinant sequences compared to all other groups of aligned sequences, using the neighbor-joining method in the K2P model [23]. The window size and step were set to 200 bp and 20 bp, respectively, with a *p*-value equal to 0.05.

## 3. Results

### 3.1. Classification Criteria of Global Historical PDCoV Strains

**Figure 1:**
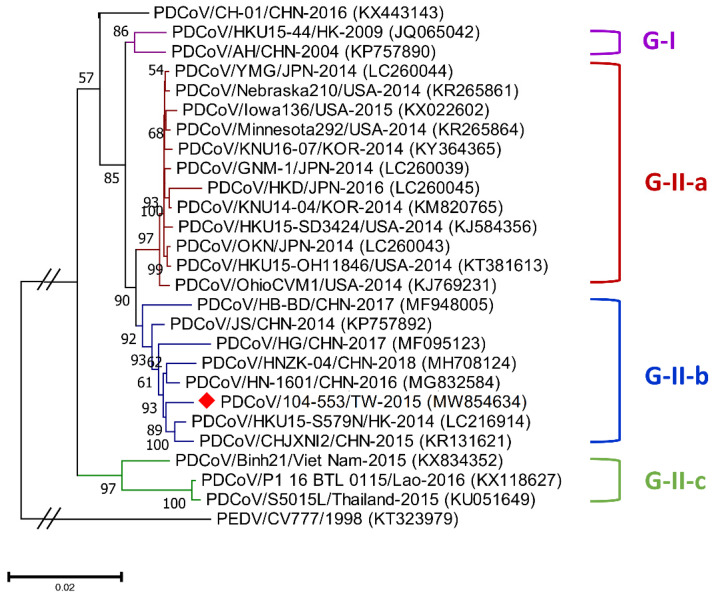
Phylogenetic trees based on the whole-genome sequencing of porcine deltacoronavirus (PDCoV) variants were established through maximum likelihood estimation (MLE), using the Kimura 2-parameter (K2P) model. PEDV/CV777/1998 served as an out-clade control. Previously identified global PDCoV strains are labeled as different genogroups in different colors on the right. The red solid diamond represents the local PDCoV isolate in 2015. The scale bar indicates nucleotide substitutions per site.

**Figure 2:**
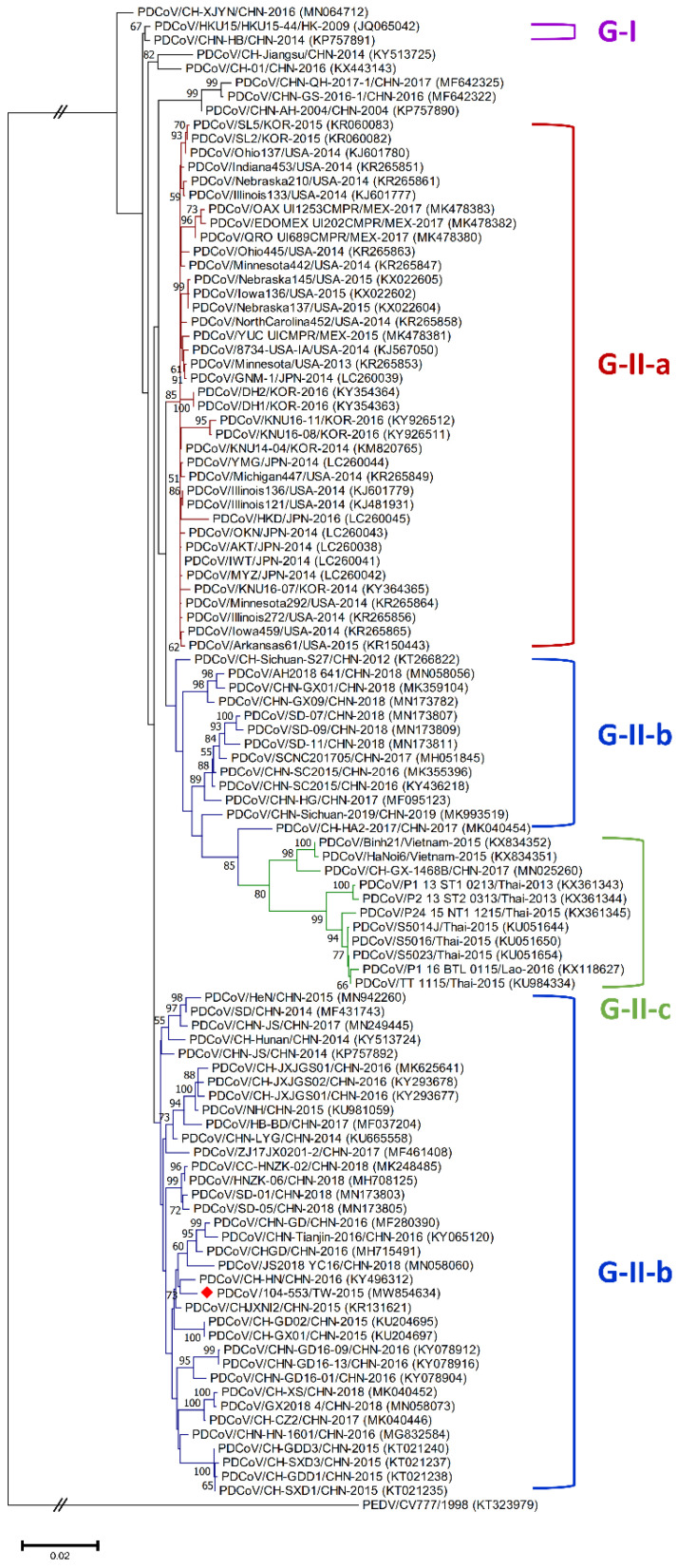
Phylogenetic trees based on the completed S gene sequences of PDCoV strains were constructed by the maximum likelihood estimation (MLE), using the Kimura 2-parameter (K2P) model. Horizontal branch lengths indicate genetic distances among various strains from different genogroups, labelled in different colors. The red solid diamond represents the local PDCoV strain in 2015. The scale bar indicates nucleotide substitutions per site.

Genetic diversities of porcine coronaviruses can be aligned and categorized by phylogeny based on the whole genome and/or S gene [13,24]. A total of 107 worldwide PDCoV variants from different countries that had outbreaks of this virus were incorporated in this study to generate effective classification criteria. Phylogenetic categorization of PDCoV sequences indicated that the virus could be separated into two main genogroups: G-I and G-II. The G-II genogroup could be further subdivided into three subgroups: G-II-a, G-II-b and G-II-c (Figure 1 and Figure 2). The G-I group included the prototype HKU15-44 and AH/CHN-2004 strains in Hong Kong and China, respectively. G-II-a PDCoV mainly involved the viral strains Minnesota292, KNU16-07, GNM-1 and OAX UI1253CMPR from the USA, Korea, Japan and Mexico, respectively. PDCoVs from China, such as HB-BD, HN-1601 and CHJXNI2, dominated the G-II-b genogroup, whereas the G-II-c subgroup was composed of variants from Southeast Asia, including Vietnam, Laos and Thailand.

### 3.2. PDCoV Genomic Sequencing

The whole PDCoV genome of strain 104-553/TW-2015 collected from intestinal specimens in Chunghua County in June 2015 was successfully sequenced and estimated to be 25,418 bp in length (GenBank accession no. MW854634). Full-length nucleotide sequences in the current study showed 98.54–98.74%, 98.50–98.93%, 98.79–99.42% and 97.22–97.80% identities to those in the G-I, G-II-a, G-II-b and G-II-c genogroups, respectively (Table 1). Regarding the S gene, the Taiwan PDCoV 104-553/TW-2015 strain manifested similar nucleotide identities of 97.14–98.70% and 97.46–98.43% to the G-I and G-II-a groups, respectively; relatively greater nucleotide identity (97.46–99.32%) to the G-II-b cluster and slightly lower nucleotide identity to G-II-c strains (95.53–96.51%). Regarding the M gene, the local PDCoV strain showed comparable sequence identity with all four genogroups, ranging from 98.44% to 99.44%. Finally, the N genes of the local PDCoV variant shared strong nucleotide identity to the G-I, G-II-a and G-II-b genogroups, fluctuating from 98.3% to 99.7%, but comparatively inferior nucleotide identity to the G-II-c strains (97.08–98.71%). The N gene of the Taiwanese PDCoV 104-553/TW-2015 strain in our study exhibited 98.40–98.61% sequence identity to the N genes of historical Taiwanese PDCoV strains [9].

### 3.3. Phylogenetic Analysis of the Whole Genome, S, M and N Genes

**Figure 3:**
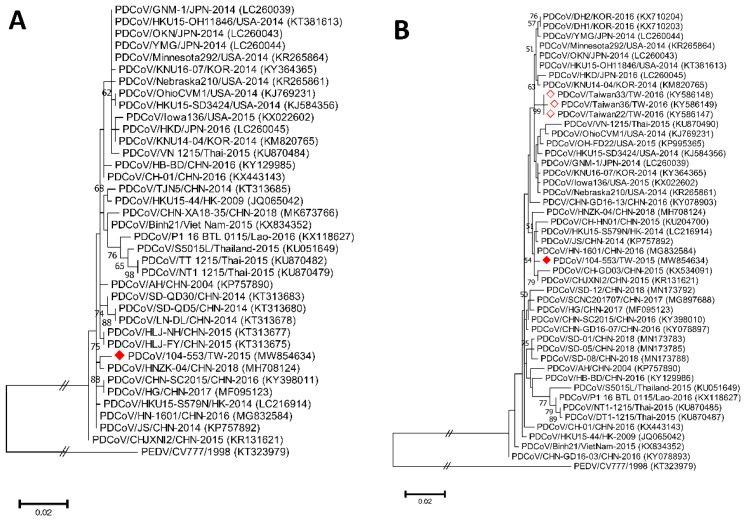
Phylogenetic trees based on the M (**A**) and N (**B**) genes of PDCoV strains were generated from maximum likelihood estimation (MLE), using the Kimura 2-parameter (K2P) model. Horizontal branch lengths represent genetic distances among various strains from different genogroups. The red solid diamond represents the local PDCoV isolate in this study. The red-outlined diamond indicates historical PDCoV strains in Taiwan.

**Figure 4:**
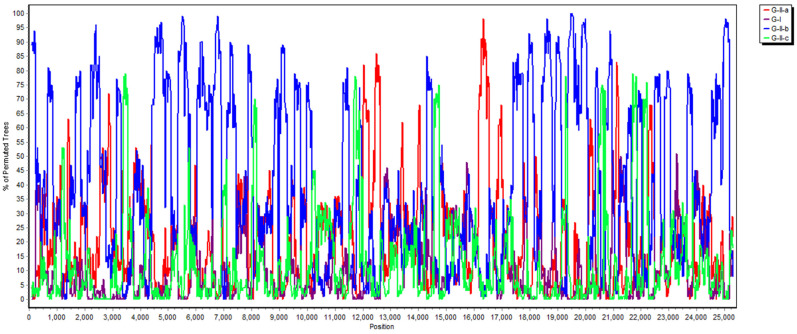
Analysis of recombination events between the local PDCoV isolate and other genogroups (G-I, G-II-a, G-II-b and G-II-c) based on the neighbor-joining method in the K2P model, using the SimPlot assay.

Phylogenetic analyses of the PDCoV/104-553/TW-2015 variant revealed that it was a member of the G-II-b clade based on both the whole genome (Figure 1) and the S gene (Figure 2). This local PDCoV variant was closely related to the Chinese strains HN-1601 and CHJXNI2, with nucleotide identities of 99.42% and 99.32%, respectively. Regarding the M and N genes, molecular phylogenies showed that the local PDCoV variant could not be effectively differentiated from other virus strains (Figure 3A,B). The recombination assay revealed no convincing event within our 104-553/TW-2015 PDCoV variant (Figure 4).

### 3.4. Amino Acid Analysis of the S Protein of the PDCoV Variant

Multiple amino acid alignments demonstrated that the PDCoV/104-553/TW-2015 strain was equipped with molecular features that were consistent with the G-II-b strains. Nevertheless, a variety of aa mutations, including V14A, F23L, P38L, S40P, Y123H, A137V, N234S, L302V, L302V, A527G, A630V, P907S and V1016I, and one specific deletion (52N) in the S protein were noted in the Taiwanese PDCoV strain, compared with one of the earliest recorded historical PDCoV strains, HKU15-44 (Table 2). In addition, three unique mutations (V551A, I670L and S698A) relative to the G-II-a strains were discovered.

## 4. Discussion

PDCoVs, which induce mild to severe diarrhea in piglets, are significant pathogens that have devastated the swine industry, since intestinal epidemics in neonatal pigs are generally correlated with PEDV [25]; however, only one study on PDCoV in Taiwan noted that PDCoV potentially existed before 2011 [9]. Genetic and phylogenic characterization of PDCoV in Taiwan has not been comprehensively performed to date. In the current study, to decipher the molecular features of PDCoV during PEDV outbreaks, we sequenced the whole genome of one PDCoV strain from a farrow-to-finish pig farm with no history of PDCoV or PEDV outbreaks. The PDCoV/104-553/TW-2015 variant was closely related to viral strains in China and was precisely assigned to the G-II-b clade. Severe diarrhea and mild vomiting were noticed in numerous piglets in this herd but with a low mortality of 5% and no coinfection with transmissible gastroenteritis virus (TGEV), PEDV or *Escherichia coli*. Additionally, numerous novel aa mutations scattered within this PDCoV sequence were identified compared to previous global strains. To the best of our knowledge, this has been the first study to report whole-genome sequencing data of PDCoV in Taiwan, and these findings serve as indispensable information for future phylogenetic studies.

Porcine coronaviruses, including TGEVs [26,27], PEDVs [13,28] and PDCoVs [29], have been thought of as highly mutating pathogens with fortuitous recombination events. For example, the G-I clade, which is characterized by the Hong Kong strains, and the G-II-b clade, which is majorly composed of Chinese strains, exhibit superior recombinant rates compared to the G-II-a cluster, which consists of strains from the USA, Mexico, Japan and Korea. Moreover, recombination hot spots have been noted in the ORF1ab gene instead of structural proteins [30]. Interestingly, recombinant events potentially occurred in some Chinese strains, such as CH-01/CHN-2016 and CH-XJYN/CHN-2016 (Figure 2), due to the cross-clade results between the phylogenies based on the whole genome and S genes. These findings represent some obstacles to molecular classification, and they probably link to the reason why PDCoV variants are intricately independent in the G-I cluster. A previous study also indicated that PDCoVs in the G-II-c lineage had dispersed to China and recombined with the original G-II-b variants [31], which explains why the G-II-b clade is discrete in Figure 1 and Figure 2. These phenomena have been noted in other coronaviruses, including type II feline coronaviruses (FCoVs) [32] and PEDVs [13]. In contrast, although no obvious recombinant event was detected in Figure 4, regions of the nucleotides (12,000, 14,000, 15,000 and between 16,000 and 17,000) located in the ORF1ab gene potentially underwent recombination (Figure 4). However, evidence indicating that the local PDCoV variant is a recombinant virus is lacking, since we cannot clearly specify the two divergent PDCoV strains from which the local variant was derived. Further exhaustive molecular characterization analyses of PDCoVs in Taiwan are urgent.

B-cell epitopes are an important factor in viral immunogenicity [33]. A previous study identified the B-cell antigenic epitope EP-4E88 (309KPKQQKKPK317) within the N protein [20], and this finding is inconsistent with the finding from our PDCoV/104-553/TW-2015 strain that highlighted a point mutation at the aa position (Pro-to-Ser [P310S]) (data not shown). As a highly conserved component of PDCoV, N protein mutations might dynamically alter the protein structure or antigenic recognition. Mutations in the specific N-glycosylation sites of the S protein might intervene in the transmissible and survival functions of the virus [34]. Furthermore, a unique deletion at aa position 52 (52N) in our PDCoV strain, which is similar to that noted in G-II-b strains, might play a crucial role in the pathogenicity of these strains in contrast to G-II-a strains. In addition, structural analysis of the adaptive evolution of PDCoV pinpoints five unique codons in the S protein (sites 107, 149, 183, 630 and 698) [30]. Compared with our strain, an aa mutation at site 630, changing from Ala (A) to Val (V), was identified. This substitute might reconstruct the hydrophobic interactions and expose Leu at position 720 of the fusion peptide region [30]. Thus, further detailed investigations of conformational dissection are needed to understand the effects of these point mutations.

## 5. Conclusions

In the present study, we filled the gap in knowledge on the molecular characterization of the full-length PDCoV genome in Taiwan during the period of prime outbreaks of PEDVs. Based on the diverse phylogenies of the current and previous global PDCoV strains, we also proposed new classification criteria, based on the whole genome and the S gene sequences. Various mutations in PDCoV might hint at potentially increased opportunities for recombination with other latently unknown PDCoVs or other porcine coronaviruses. Future studies should focus on continuously monitoring the genetic diversity and mutations in PDCoV and simultaneously launch applicable preventive strategies against this problematic virus.

## Figures and Tables

**Table 1:** Nucleotide identities (%) among porcine deltacoronavirus strains from Taiwan and four different genogroups defined in the present study.

PDCoV/104-553/ TW-2015	Genogroups				
	G-I	G-II-a	G-II-b	G-II-c	Taiwan Strains
Full-length genome	98.54–98.74	98.50–98.93	98.79–99.42	97.22–97.80	-
S gene	97.14–98.70	97.46–98.43	97.46–99.32	95.53–96.51	-
M gene	99.16–99.44	98.88–99.16	98.87–99.44	98.44–99.30	-
N gene	98.30–98.91	98.50–99.01	98.50–99.70	97.08–98.71	98.40–98.61

**Table 2:** Comparison of amino acid mutations of the S protein among the Taiwan 104-553 strain and historical reference strains from each genogroup. PDCoV strains represented the G-I (HKU15-44/HK-2009), undetermined strain (CHN-HB/CHN-2014 strain to CHN-AH-2004/CHN-2004 strain), G-II-a (Iowa136/USA-2015 strain to YUC_UICMPR/MEX-2015 strain), G-II-b (CHN-HN-1601/CHN-2016 strain to CH-HA2-2017/CHN-2017), and G-II-c (Binh21/Vietnam-2015 to P1_16_BTL_0115/Lao-2016) genogroups, respectively. Dots indicate the analogous amino acids. Dashes denote deletions.

	Mutations and/or Deletions of Amino Acids															
	S1 Region											S2 Region				
PDCoV Strains	14	23	38	40	52	123	137	234	302	527	551	630	670	698	907	1016
HKU15-44/HK-2009	V	F	P	R	N	Y	A	N	L	A	A	A	L	A	P	V
CHN-HB/CHN-2014	.	L	.	.	-	.	.	I	.	.	.	.	.	.	.	.
CH-01/CHN-2016	.	L	L	S	-	.	.	S	.	.	.	.	.	.	S	.
CHN-QH-2017-1/CHN-2017	.	L	.	.	.	.	.	S	.	.	.	.	V	S	S	.
CHN-AH-2004/CHN-2004	.	L	.	.	.	.	.	S	.	.	.	.	V	S	S	.
Iowa136/USA-2015	.	L	.	.	.	.	.	R	.	.	V	.	I	S	S	I
Nebraska210/USA-2014	.	L	.	.	.	.	.	S	.	.	V	.	I	S	S	I
Ohio137/USA-2014	.	L	.	.	.	.	.	S	.	.	V	.	I	S	S	I
Indiana453/USA-2014	.	L	.	.	.	.	.	S	.	.	V	.	I	S	S	I
Minnesota292/USA-2014	.	L	.	.	.	.	.	S	.	.	V	.	I	S	S	I
Michigan447/USA-2014	.	L	.	.	.	.	.	S	.	.	V	.	I	S	S	I
HKD/JPN-2016	.	L	.	.	.	.	.	S	.	.	V	.	I	S	S	I
AKT/JPN-2014	.	L	.	.	.	.	.	S	.	.	V	.	I	S	S	I
KNU14-04/KOR-2014	.	L	.	.	.	.	.	S	.	.	V	.	I	S	S	I
HKU14-11/KOR-2016	.	.	.	.	.	.	.	S	.	.	V	.	I	S	S	I
YUC_UICMPR/MEX-2015	.	L	.	.	.	.	.	S	.	.	V	.	I	S	S	I
CHN-HN-1601/CHN-2016	.	L	L	S	-	.	V	S	.	.	.	.	I	.	S	I
CH-XS/CHN-2018	.	L	L	S	-	.	V	S	.	.	.	L	I	S	S	.
CHJXNI2/CHN-2015	.	L	L	S	-	.	V	S	.	.	.	.	.	.	S	I
CH-GX01/CHN2015	.	L	L	S	-	.	V	S	.	.	.	.	.	.	S	.
CH-HN/CHN-2016	.	L	L	S	-	.	V	S	.	.	.	.	.	.	S	I
HB-BD/CHN-2017	.	L	L	S	-	H	.	S	.	.	.	.	.	.	S	I
AH2018_641/CHN-2018	.	L	L	S	-	.	.	S	.	.	V	.	.	.	.	.
CHN-SC2015/CHN-2016	.	L	L	S	-	.	.	S	.	.	V	.	I	.	S	.
CHN-HG/CHN-2017	.	L	L	S	-	.	.	S	.	.	V	.	I	.	S	.
CH-HA2-2017/CHN-2017	.	L	L	S	-	.	V	S	.	.	V	.	V	S	T	.
Binh21/Vietnam-2015	A	L	.	.	.	.	.	S	.	.	V	.	V	S	.	.
P1_13_ST1_0213/Thai-2013	A	L	.	.	.	H	.	S	.	.	V	.	V	S	.	.
S5016/Thai-2015	A	L	.	.	.	.	.	S	.	.	.	.	V	S	.	.
P1_16_BTL_0115/Lao-2016	.	L	.	.	.	.	.	S	.	.	.	.	V	S	.	.
104-553/TW-2015	A	L	L	S	-	H	V	S	V	G	.	V	.	.	S	I

## Data Availability

Not applicable.

## Supplementary Materials

The following is available online at https://www.mdpi.com/article/10.3390/v13071337/s1, Table S1: Information on the 17 primer pairs used in the present study.
