# Supplementary material for: Phylogenetic Classification of Global Porcine Deltacoronavirus (PDCoV) Reference Strains and Molecular Characterization of PDCoV in Taiwan
Source: Viruses. 2021 Jul 11;13(7):1337. doi: 10.3390/v13071337 (PMC8310012; doi:10.3390/v13071337)
Supplement: Supplementary file 1 [file viruses-13-01337-s001.zip › viruses-1287284-supplementary.pdf]

Supplementary material

Table S1. Information on the 17 primer pairs used in the present study.

Primer	Sequences (5′-3′)	Nucleotide position	Annealing temperature (°C)	Reference
DCor-F1	ACATGGGGACTAAAGATAAAAATTATAGC	1-1603	62	[2]
DCor-R1	AGACGGGCCAATTTTGACCG			
DCor-F2	TGATGATGTTCTGCTAGCCT	1474-3293	61	[2]
DCor-R2	GCTCATCGCCTACATCAGTA			
DCor-F3	CGGATTTAAAACCACAGACT	3084-4853	58	[2]
DCor-R3	ACGACTTTACGAGGATGAAT			
DCor-F4	CTCCTGTACAGGCCTTACAA	4734-6413	61	[2]
DCor-R4	TCACACGTATAGCCTGCTGA			
DCor-F5	CTCAATGCAGAAGACCAGTC	6284-8053	59	[2]
DCor-R5	CAGCTTGGTCTTAAGACTCT			
DCor-F6	GGTACTGCTTCTGATAAGGAT	7913-9653	59	[2]
DCor-R6	TAGGTACAGTTGTGAACCGA			
DCor-F7	CTCTGCCCATTATCATGCCT	9534-11033	61	[2]
DCor-R7	AAAGAGAGGCATTTTGCTGG			
DCor-F8	ACTTGGACCCTCCTATGCGC	10854-12833	58	[2]
DCor-R8	GGCTCAAGATACTTATCTGC			
DCor-F9	TGCAGGATGGTGAAGC	12716-13724	58	This study
DCor-R9	CCATCAAGATCAGCAACAG			
DCor-F10	CTGCTAATGTAGCCACCT	13484-14465	58	This study
DCor-R10	GCAGTGTGGCGATAGA			
DCor-F11	TGTTACGCAGACTACACATA	14274-16013	58	[2]
DCor-R11	TCATAGCCGCAGCGCTTAAA			
DCor-F12	TGTGGTGTTTAGGCAGGCAA	15894-17753	64	[2]
DCor-R12	GTGGCGGTTACGCCTAAACC			
DCor-F13	CAAACTCTTTGACAATCGCA	17633-19193	59	[2]
DCor-R13	GCTAAAGGAGAATAGGTTGGTG			
DCor-F14	CTGAACATTCATTCTCACCC	18974-20906	58	[2]
DCor-R14	GAAGGTGGTGGCATTTGTGG			
DCor-F15	GTCTTACCGTGTGAAACCCC	20757-22436	63	[2]
DCor-R15	AACATCCCACTGAGGAGGTG			
DCor-F16	TTTTATAACACCACCGCTGC	22317-24000	60	[2]
DCor-R16	GGCCATGATAGATTGGTGTC			
DCor-F17	ATGGTGAGCCTTTACTGCTT	23877-25415	62	[2]
DCor-R17	TGCTCCATCCCCCCTATAAG			

*Nucleotide positions are based on the reference porcine coronavirus HKU15-44 strain (GenBank accession no. JQ065042)
